# Posttraumatic Stress Disorder Symptoms Among First-Year Resident Physicians Working Before and During the COVID-19 Pandemic

**DOI:** 10.1001/jamanetworkopen.2023.30241

**Published:** 2023-08-22

**Authors:** Michelle K. Ptak, Elena Frank, Katherine E. T. Ross, Jennifer L. Cleary, Srijan Sen, Karina Pereira-Lima

**Affiliations:** 1 Department of Psychology, University of Michigan, Ann Arbor; 2 Michigan Neuroscience Institute, University of Michigan Medical School, Ann Arbor; 3 Eisenberg Family Depression Center, University of Michigan Medical School, Ann Arbor; 4 Department of Neurology, University of Michigan Medical School, Ann Arbor

## Abstract

This cohort study investigates differences in posttraumatic stress disorder (PTSD) symptoms among first-year resident physicians training before and during the first wave of the COVID-19 pandemic (March to June 2020).

## Introduction

During the COVID-19 pandemic, resident physicians experienced rapidly changing training environments, raising concerns about their risk of trauma exposure and posttraumatic stress disorder (PTSD).^1^ Although cross-sectional studies^2,3^ reported a high prevalence of PTSD symptoms among residents during the pandemic, to our knowledge, no studies have assessed whether this prevalence differs from prepandemic levels. Here, we investigated differences in PTSD symptoms among first-year residents training before and during the first pandemic wave (March to June 2020), while accounting for established factors associated with mental health outcomes^4,5^ in training physicians.

## Methods

This cohort study follows the Strengthening the Reporting of Observational Studies in Epidemiology (STROBE) reporting guideline and was approved by the University of Michigan Review Board. Participants provided electronic informed consent and were compensated $125. We analyzed data from 2 cohorts (2018-2019 [prepandemic] and 2019-2020 [during pandemic]) of the Intern Health Study,^4,5^ a longitudinal cohort study of first-year resident physicians. Participants completed a baseline survey 2 months before their residency start, followed by quarterly surveys during the intern year (eAppendix 1 in Supplement 1). The fourth quarterly survey included the adapted Primary Care PTSD Screen for *Diagnostic and Statistical Manual of Mental Disorders* (Fifth Edition) (PC-PTSD-5).^5^

Consistent with previously described methods,^4^ we used poststratification and attrition weights to reduce nonrepresentative sampling and participation attrition biases (eAppendix 2 in Supplement 1). Differences in nonresidency factors and residency-related factors before and during the pandemic were assessed via χ^2^ and independent *t* tests. Associations between positive PTSD screen (PC-PTSD-5 score ≥3) and study cohort (prepandemic vs during pandemic) among all participants and those exposed to workplace trauma (ie, traumatic events experienced at work) were assessed using univariable and multivariable logistic regression models accounting for nonresidency and residency-related factors (significance was set at 2-sided *P* < .05). Analyses were conducted in R statistical software version 4.2.2 (R Project for Statistical Computing).

## Results

Of 3814 residents enrolled in the parent study, 1957 (51.3%) completed the PC-PTSD-5 and were included (943 [48.2%] female; mean [SD] age, 27.6 [2.6] years; 1137 [58.1%] prepandemic cohort; 820 [41.9%] during pandemic cohort). Among nonresidency factors, only neuroticism was significantly higher among residents training during the pandemic vs prepandemic residents (score mean difference [MD], 0.9; 95% CI, 0.1 to 1.7). Among residency-related factors, residents training during the pandemic reported significantly lower weekly duty hours (MD, −3.1 hours; 95% CI, −4.1 to −2.0 hours), lower mean reports of medical errors (score MD, −0.04; 95% CI, −0.06 to −0.01), and higher workload satisfaction (score MD, 0.2; 95% CI, 0.2 to 0.3) (Table 1).

**Table 1.** Non–Residency-Related and Residency-Related Characteristics of Participants

Variable	Participants, No. (%)					
	Weighted sample			Nonweighted sample		
	Prepandemic cohort (n = 1137)	During pandemic cohort (n = 820)	*P* value^a^	Prepandemic cohort (n = 1137)	During pandemic cohort (n = 820)	*P* value^a^
Non–residency-related factors assessed at baseline						
Gender						
Male	584 (51.3)	430 (52.5)	.62	471 (41.4)	371 (45.2)	.09
Female	553 (48.7)	390 (47.5)		666 (58.6)	449 (54.8)	
Sexual orientation						
Heterosexual	1027 (90.3)	718 (87.6)	.05	1026 (90.2)	720 (87.8)	.09
Lesbian, gay, bisexual, or any other not specified	110 (9.7)	102 (12.4)		111 (9.8)	100 (12.2)	
Age, mean (SD), y	27.5 (2.5)	27.7 (2.7)	.09	27.5 (2.6)	27.6 (3.2)	.43
Race and/or ethnicity						
Underrepresented in medicine^b^	227 (20.0)	182 (22.2)	.23	199 (17.5)	157 (19.1)	.35
Not underrepresented in medicine	906 (79.7)	635 (77.4)		936 (82.3)	661 (80.6)	
Missing	3 (0.3)	3 (0.4)		2 (0.2)	2 (0.2)	
Marital status						
Married	314 (27.6)	204 (24.9)	.17	311 (27.4)	202 (24.6)	.18
Unmarried	823 (72.4)	616 (75.1)		826 (72.6)	618 (75.4)	
Parental status						
Has children	83 (7.3)	65 (7.9)	.57	79 (6.9)	61 (7.4)	.68
Does not have children	1054 (92.7)	755 (92.1)		1058 (93.1)	759 (92.6)	
Risky Families Questionnaire score, mean (SD)	12.7 (9.5)	12.8 (9.4)	.38	12.7 (9.2)	12.8 (9.3)	.66
Neuroticism score (NEO Five-Factor Inventory), mean (SD)	22.9 (8.6)	23.7 (8.8)	.04	22.6 (8.5)	23.5 (8.8)	.03
History of depression						
Yes	537 (47.2)	379 (46.2)	.65	544 (47.8)	388 (47.3)	.82
No	600 (52.8)	441 (53.8)		593 (52.2)	432 (52.7)	
Non–residency-related factor assessed at quarterly surveys						
Cumulative exposure to stressful life events, mean (SD)^c^	0.21 (0.26)	0.21 (0.27)	.79	0.21 (0.26)	0.21 (0.27)	.91
Residency-related factor assessed at baseline						
Specialty						
Surgical	213 (18.8)	143 (17.5)	.46	239 (21.0)	157 (19.1)	.31
Nonsurgical	924 (81.2)	677 (82.5)		898 (79.0)	663 (80.9)	
Residency-related factors assessed at quarterly surveys						
Weekly duty hours, mean (SD)	63.5 (12.5)	60.2 (11.9)	<.001	63.1 (12.1)	60.0 (11.6)	<.001
Cumulative reports of medical errors, mean (SD)^d^	0.22 (0.30)	0.19 (0.30)	.01	0.23 (0.30)	0.19 (0.28)	<.01
Workload satisfaction–Resident Questionnaire score, mean (SD)^e^	3.20 (0.70)	3.44 (0.67)	<.001	3.21 (0.69)	3.44 (0.66)	<.001
Learning environment satisfaction–Resident Questionnaire score, mean (SD)^e^	3.73 (0.58)	3.76 (0.56)	.19	3.73 (0.57)	3.77 (0.56)	.19

^a^ *P* values for comparisons were calculated using the χ^2^ test for categorical variables and using the *t* test for numerical variables.

^b^ Residents were coded as underrepresented according to the American Association of Medical Colleges definition as “racial and ethnic populations that are underrepresented in the medical professional relative to their numbers in the general population” (eReferences in Supplement 1). In this study, this group included residents self-identifying as African American, Arab or Middle Eastern, Latino, Native American, Pacific Islander, other, or multiracial. Residents’ race and ethnicity data were collected to assign appropriate sample weights based on the demographic distribution of the overall population of US first-year resident physicians.

^c^ Cumulative scores in the stressful life events scale (eAppendix 1 in Supplement 1) in each quarterly assessment were averaged to obtain the mean cumulative exposure to these events during the first residency year.

^d^ Responses to the medical error question (eAppendix 1 in Supplement 1) in each quarter were scored as yes (1) and no (0). Scores in each quarterly assessment were averaged to obtain the mean score of reports of medical errors during the first residency year.

^e^ The workload satisfaction and learning environment scales of the Resident Questionnaire are assessed only at the last quarterly survey at month 12 of the residency year 1.

Residents training during the pandemic were significantly less likely than prepandemic residents to screen positive for PTSD (58 [7.1%] vs 122 [10.7%] residents; odds ratio [OR], 0.64; 95% CI, 0.46-0.88; *P* = .01) and workplace trauma exposure (418 [50.9%] vs 643 [56.6%] residents; OR, 0.80; 95% CI, 0.66-0.95; *P* = .01). In multivariable models accounting only for nonresidency factors, training during the pandemic remained significantly associated with lower odds of screening positive for PTSD (OR, 0.59; 95% CI, 0.42-0.82). After incorporating residency-related factors, there was no statistically significant association between training during the pandemic and the odds of presenting PTSD symptoms (OR, 0.76; 95% CI, 0.53-1.09) (Table 2).

**Table 2.** Logistic Regression Models of Positive Screen for Posttraumatic Stress Disorder Among All Participants and Participants Exposed to Workplace Trauma

Variables	All participants (N = 1957)				Participants exposed to workplace trauma (n = 1061)			
	Weighted models		Nonweighted models		Weighted models		Nonweighted models	
	Odds ratio (95% CI)	*P* value	Odds ratio (95% CI)	*P* value	Odds ratio (95% CI)	*P* value	Odds ratio (95% CI)	*P* value
Univariable model								
Study cohort, during pandemic cohort (reference: prepandemic cohort)	0.64 (0.46-0.88)	.01	0.66 (0.47-0.90)	.01	0.69 (0.49-0.97)	.04	0.70 (0.50-0.98)	.04
Model accounting only for non–residency-related factors								
Study cohort, during pandemic cohort (reference: prepandemic cohort)	0.59 (0.42-0.82)	.002	0.61 (0.43-0.85)	.004	0.66 (0.46-0.95)	.03	0.67 (0.47-0.95)	.03
Non–residency-related factors								
Female (reference: male)	1.46 (1.05-2.04)	.03	1.41 (1.00-2.00)	.05	1.39 (0.98-1.98)	.06	1.35 (0.94-1.96)	.10
Age (for each 1-y increase)	0.97 (0.91-1.02)	.22	0.96 (0.91-1.01)	.12	0.97 (0.92-1.03)	.43	0.97 (0.92-1.03)	.29
Lesbian, gay, bisexual, or any other not specified (reference: heterosexual)	1.74 (0.73-1.84)	.50	1.24 (0.77-1.93)	.35	1.11 (0.66-1.79)	.69	1.15 (0.69-1.84)	.59
Underrepresented in medicine (reference: nonunderrepresented)^a^	1.26 (0.87-1.81)	.22	1.26 (0.85-1.82)	.24	1.28 (0.86-1.88)	.21	1.26 (0.84-1.88)	.26
Married (reference: unmarried)	0.70 (0.45-1.07)	.11	0.76 (0.49-1.14)	.20	0.69 (0.44-1.07)	.11	0.74 (0.47-1.14)	.19
No children (reference: has children)	1.08 (0.51-2.45)	.84	1.07 (0.52-2.42)	.86	1.23 (0.53-2.61)	.76	1.06 (0.49-2.45)	.89
History of depression (reference: no history of depression)	1.85 (1.32-2.63)	.001	1.72 (1.23-2.43)	.002	1.64 (1.14-2.38)	.01	1.54 (1.08-2.22)	.02
Risky early family environment (for each 1-point increase)	1.02 (1.01-1.04)	.01	1.02 (1.01-1.04)	.01	1.01 (1.00-1.03)	.09	1.01 (1.00-1.03)	.11
Neuroticism (for each 1-point increase)	1.03 (1.01-1.05)	.01	1.03 (1.01-1.05)	.001	1.03 (1.01-1.05)	.01	1.04 (1.01-1.06)	.002
Mean cumulative exposure to stressful life events (for each 1-point increase)^b^	4.69 (2.73-8.02)	<.001	4.80 (2.83-8.14)	<.001	4.40 (2.46-7.89)	<.001	4.44 (2.51-7.86)	<.001
Model accounting for both non–residency-related and residency-related factors								
Study cohort, during pandemic cohort (reference: prepandemic cohort)	0.76 (0.53-1.09)	.15	0.78 (0.55-1.11)	.18	0.83 (0.56-1.20)	.32	0.82 (0.56-1.19)	.30
Non–residency-related factors								
Female (reference: male)	1.65 (1.17-2.35)	.01	1.59 (1.11-2.30)	.01	1.57 (1.09-2.29)	.02	1.51 (1.03-2.22)	.04
Age (for each 1-y increase)	0.96 (0.90-1.01)	.11	0.95 (0.90-1.00)	.06	0.95 (0.90-1.01)	.12	0.95 (0.90-1.01)	.07
Lesbian, gay, bisexual, or any other not specified (reference: heterosexual)	1.04 (0.63-1.68)	.87	1.10 (0.67-1.76)	.69	0.92 (0.54-1.54)	.76	0.97 (0.57-1.61)	.92
Underrepresented in medicine (reference: nonunderrepresented)^a^	1.30 (0.88-1.90)	.18	1.26 (0.84-1.86)	.25	1.25 (0.83-1.88)	.28	1.23 (0.80-1.87)	.33
Married (reference: unmarried)	0.68 (0.42-1.07)	.10	0.75 (0.47-1.15)	.20	0.68 (0.42-1.09)	.11	0.74 (0.46-1.17)	.21
No children (reference: has children)	0.83 (0.39-1.93)	.65	0.85 (0.40-1.97)	.70	0.79 (0.36-1.90)	.59	0.78 (0.35-1.85)	.55
History of depression (reference: no history of depression)	1.66 (1.16-2.38)	.01	1.59 (1.11-2.27)	.01	1.66 (1.14-2.44)	.01	1.54 (1.06-2.27)	.02
Risky early family environment (for each 1-point increase)	1.01 (1.00-1.03)	.10	1.02 (1.00-1.03)	.07	1.01 (0.99-1.03)	.44	1.01 (0.99-1.02)	.44
Neuroticism (for each 1-point increase)	1.02 (0.99-1.04)	.14	1.02 (1.00-1.04)	.05	1.02 (1.00-1.04)	.08	1.03 (1.01-1.05)	.02
Mean cumulative exposure to stressful life events (for each 1-point increase)^b^	4.29 (2.40-7.65)	<.001	4.40 (2.49-7.72)	<.001	3.84 (2.07-7.13)	<.001	3.82 (2.08-7.00)	<.001
Residency-related factors								
Surgical specialty (reference: nonsurgical specialty)	0.47 (0.28-0.77)	.003	0.45 (0.27-0.72)	.001	0.44 (0.25-0.74)	.002	0.43 (0.25-0.71)	.001
Mean cumulative reports of medical errors (for each 1-point increase)^c^	4.75 (2.91-7.72)	<.001	4.28 (2.62-6.96)	<.001	3.70 (2.19-6.25)	<.001	3.29 (1.95-5.55)	<.001
Weekly work hours (for each 1-point increase)	1.02 (1.01-1.05)	.001	1.03 (1.01-1.04)	.002	1.03 (1.01-1.05)	.004	1.02 (1.01-1.04)	.01
Workload satisfaction – Resident Questionnaire (for each 1-point increase)	0.57 (0.43-0.76)	<.001	0.57 (0.43-0.75)	<.001	0.61 (0.45-0.83)	.002	0.60 (0.44-0.82)	.001
Learning environment satisfaction–Resident Questionnaire (for each 1-point increase)	0.84 (0.60-1.17)	.29	0.85 (0.61-1.18)	.32	0.75 (0.53-1.08)	.12	0.78 (0.54-1.10)	.16

^a^ Residents were coded as underrepresented using the American Association of Medical Colleges definition as “racial and ethnic populations that are underrepresented in the medical profession relative to their numbers in the general population” (eReferences in Supplement 1). Here, this group included residents self-identifying as African American, Arab/Middle Eastern, Latino, Native American, Pacific Islander, other, or multiracial. Residents’ race and ethnicity data were collected to assign appropriate sample weights based on the demographic distribution of the overall population of US first-year resident physicians.

^b^ Cumulative scores in the stressful life events scale (eAppendix 1 in Supplement 1) in each quarterly assessment were averaged to obtain the mean cumulative exposure to these events during the first residency year.

^c^ Responses to the medical error question (eAppendix 1 in the Supplement) in each quarter were scored as yes (1) and no (0). Scores in each quarterly assessment were averaged to obtain the mean score of reports of medical errors during the first residency year.

## Discussion

In this cohort study, compared with those training before the pandemic, first-year residents training during the first pandemic wave were significantly less likely to screen positive for PTSD and workplace trauma exposure. Additionally, they reported significantly fewer weekly work hours, higher workload satisfaction, and fewer medical errors, which could reflect previously reported institutional efforts to reduce physician burden early in the pandemic.^6^ Importantly, after accounting for these residency-related factors, training during the pandemic was no longer associated with lower odds of presenting PTSD symptoms.

This study builds on prior work^2,3^ by demonstrating that, although the prevalence of PTSD symptoms among residents was high during the pandemic, it was significantly lower than prepandemic levels. In addition, these findings identify work hours, workload, and medical errors as potential targets of intervention to prevent PTSD among residents. Limitations include the study’s self-reported nature; its restriction to the first pandemic wave, first-year residents, and prepandemic data for a single academic year; general decreases in survey participation during the pandemic; and possible unmeasured factors associated with PTSD risk. Future studies should follow residents’ PTSD symptoms after the first pandemic wave and investigate whether interventions targeting the identified residency-related factors could reduce their PTSD risk moving forward.

## Notes

Supplement 1. **eAppendix 1.** Recruitment Strategy and Survey Questions Used in the Present Study **eAppendix 2.** Survey Weights Strategy
eReferences

Supplement 2.
Data Sharing Statement

## References

[1] d’Ettorre G, Ceccarelli G, Santinelli L, . Post-traumatic stress symptoms in healthcare workers dealing with the COVID-19 pandemic: a systematic review. Int J Environ Res Public Health. 2021;18(2):601. doi:10.3390/ijerph18020601 33445712 PMC7828167

[2] Chang J, Ray JM, Joseph D, Evans LV, Joseph M. Burnout and post-traumatic stress disorder symptoms among emergency medicine resident physicians during the COVID-19 pandemic. West J Emerg Med. 2022;23(2):251-257. doi:10.5811/westjem.2021.11.53186 35302461 PMC8967473

[3] Ghahramani S, Kasraei H, Hayati R, Tabrizi R, Marzaleh MA. Health care workers’ mental health in the face of COVID-19: a systematic review and meta-analysis. Int J Psychiatry Clin Pract. 2023;27(2):208-217. doi:10.1080/13651501.2022.2101927 35875844

[4] Fang Y, Bohnert ASB, Pereira-Lima K, . Trends in depressive symptoms and associated factors during residency, 2007 to 2019: a repeated annual cohort study. Ann Intern Med. 2022;175(1):56-64. doi:10.7326/M21-1594 34781718

[5] Vance MC, Mash HBH, Ursano RJ, . Exposure to workplace trauma and posttraumatic stress disorder among intern physicians. JAMA Netw Open. 2021;4(6):e2112837. doi:10.1001/jamanetworkopen.2021.12837 34100937 PMC8188264

[6] Sinsky C, Linzer M. Practice and policy reset post-COVID-19: reversion, transition, or transformation? Health Aff (Millwood). 2020;39(8):1405-1411. doi:10.1377/hlthaff.2020.00612 32744939

