# Supplementary material for: Posttraumatic Stress Disorder Symptoms Among First-Year Resident Physicians Working Before and During the COVID-19 Pandemic
Source: JAMA Netw Open. 2023 Aug 22;6(8):e2330241. doi: 10.1001/jamanetworkopen.2023.30241 (PMC10445197; doi:10.1001/jamanetworkopen.2023.30241)
Supplement: Supplement 1. — eAppendix 1. Recruitment Strategy and Survey Questions Used in the Present Study eAppendix 2. Survey Weights Strategy eReferences [file jamanetwopen-e2330241-s001.pdf]

Supplemental Online Content

Ptak MK, Frank E, Ross KET, Cleary JL, Sen S, Pereira-Lima K. Posttraumatic stress disorder symptoms among first-year resident physicians working before and during the COVID-19 pandemic. *JAMA Netw Open*. 2023;6(8):e2330241. doi:10.1001/jamanetworkopen.2023.30241

eAppendix 1. Recruitment Strategy and Survey Questions Used in the Present Study
eAppendix 2. Survey Weights Strategy
eReferences

This supplemental material has been provided by the authors to give readers additional information about their work.

© 2023 Ptak MK et al. *JAMA Network Open.*

eAppendix 1. Recruitment Strategy and Survey Questions Used in the Present Study

Recruitment strategy

The present study was performed as part of the Intern Health Study,[1] a larger longitudinal cohort study that assesses the role of psychological, biological, and environmental factors in the development of depression under stress. Following the 2018-2019 and 2019-2020 academic year national resident match, email addresses for incoming first-year residents across all specialties throughout the United States were gathered from residency programs and publicly available databases. Eligible residents (ie, incoming first-year resident physicians in US residency programs that offer graduate year 1 positions available immediately after medical school completion) were invited via email to complete a web-based baseline confidential survey two months before beginning their first year of residency (ie, internship year) and four quarterly surveys during their internship year. The survey questions used in the present study are presented below.

Baseline survey questions used in the present study

Demographic Questions

- Date of Birth (MM/DD/YYYY)

- Gender
  ( )Male
  ( )Female

- Ethnicity (check all that apply)
  ( )White
  ( )Black / African American
  ( ) Latino / Hispanic
  ( ) Asian (e.g. Indian, Chinese)
  ( ) Arab / Middle Eastern
  ( ) Native American
  ( ) Pacific Islander
  ( ) Other ____________________

- Which of the following best describes your sexual orientation?
  ( ) Heterosexual
  ( ) Gay / Lesbian
  ( ) Bisexual
  ( ) Other ____________________
  ( ) Prefer not to say

- Current marital status
  ( ) Single

© 2023 Ptak MK et al. *JAMA Network Open.*

( ) Engaged
( ) Married
( ) Separated / Divorced

- Do you have a child or children?
  ( ) Yes
  ( ) No

Risky Family Environment: Risky Families Questionnaire[2]

Risky early family environment was assessed using the 13-item Risky Families Questionnaire,[2] in which participants retrospectively self-reported any abuse, neglect, or family conflict occurring between ages 5 and 15. Each item ranges from 1 (not at all) to 5 (very often), with higher scores denoting a more adverse childhood.

Neuroticism: Neuroticism scale of the NEO-Five-Factor Inventory[3]

Neuroticism was measured using the neuroticism subscale of the NEO-Five Factor Inventory.[3] Each item presents a statement, such as “I rarely feel anxious or nervous” or “I often worry about things that might go wrong”, that participants respond to using a five-point Likert scale ranging from “0 = strongly disagree” to “4 = strongly agree.” Higher scores indicate higher levels of neuroticism.

History of Depression

- To the best of your recollection, have you EVER experienced an episode of depression (a two week period of your life when you felt down or lost interest or pleasure in your usual activities and also had difficulty concentrating or noticed changes in sleep, appetite, energy or experienced thoughts of death or feelings of guilt)?
  ( ) Yes
  ( ) No

Specialty (2018 cohort)

- Residency specialty
  ( ) Internal Medicine
  ( ) Surgery
  ( ) Obstetrics/Gynecology
  ( ) Pediatrics
  ( ) Psychiatry
  ( ) Emergency Medicine
  ( ) Med/Peds
  ( ) Family Medicine
  ( ) Anesthesiology (without transitional year)
  ( ) Neurology (without transitional year)
  ( ) Otolaryngology (without transitional year)
  ( ) Transitional Year

( ) Other____________________

Specialty (2019 cohort)

In 2019, the Intern Health Study started using specialty information from recruitment and removed the specialty question from baseline.

<u>Quarterly survey questions used in the present study</u>

Duty Hours (assessed at months 3, 6, 9, and 12 of internship year)

- How many hours have you worked in the PAST WEEK? _________

Recent Stressful Life Events (assessed at months 3, 6, 9, and 12 of internship year)

Recent Stressful Life Events (SLEs) were assessed using the following 11-item scale developed by the Intern Health Study.[1] Items inquire about a series of SLEs, yielding a score of 1 at each time the respondent endorses any item, for a total score ranging from 0 to 11 points. For the purposes of the present study, cumulative scores in the Recent Stressful Life Events scale in each quarterly survey assessment were averaged in order to obtain the mean cumulative exposure to SLEs during the internship year.

- Please indicate if you have experienced any of the following events during the PAST 3 MONTHS (select all that apply).

( ) Death of a family member, significant other or close friend
( ) You developed a disabling illness or injury lasting a month or more
( ) A disabling physical illness or injury started or got worse in a family member, significant other or close friend
( ) A relationship with an intimate cohabiting partner ended
( ) You were involved in a physically violent relationship
( ) You suffered a significant financial loss or loss of property
( ) You had problems with debt i.e., having items repossessed, not having enough money to pay household expenses, lacking money for medical expenses or difficulty paying bills
( ) You were physically assaulted or attacked
( ) You got married
( ) You learned that you or your partner were pregnant
( ) You had a child

Medical Errors (assessed at months 3, 6, 9, and 12 of internship year)

For the purposes of the present study, we obtained the mean cumulative reports of medical errors by averaging residents' scores (0="no", 1="yes") reported for the question below in each quarterly assessment.

- Are you concerned you have made any major medical errors in the LAST 3 MONTHS?

(1) Yes
(0) No

© 2023 Ptak MK et al. *JAMA Network Open.*

Workload Satisfaction and Learning Environment Satisfaction (assessed at month 12 of internship year): Workload and Learning Environment scales of the Resident Questionnaire[4]

Resident physicians' satisfaction with their program workload and learning environment was assessed using the workload (8 items) and learning environment satisfaction (9 items) subscales of the Resident Questionnaire.[4] The Resident Questionnaire is a valid measure of medical residents' satisfaction with various aspects of their program relating to both workload and learning environments, such as caseload, time demands, faculty feedback, and personal support. For each item, respondents are asked to indicate whether they agree with the presented statement using a five-point Likert scale ranging from "1 = strongly agree" to "5 = strongly disagree." Higher scores indicate higher levels of satisfaction with the program workload and learning environment.

Workplace trauma exposure and PTSD Symptoms (assessed at month 12 of internship year): Adapted Primary Care PTSD Screen for DSM-5 (PC-PTSD-5)[5,6]

The PC-PTSD-5 scale[5] is a 5-item questionnaire that screens for a probable PTSD diagnosis based on DSM-5 diagnostic criteria. The first part of the scale, which screens for workplace trauma exposure, has been previously adapted[6] to specifically screen for healthcare-related trauma that occurred in the workplace: "Sometimes things happen to physicians that are unusually or especially frightening, horrible, or traumatic. For example: sudden patient deaths, serious medical errors, workplace violence, hazardous exposure, or repeated or extreme exposure to the details of traumatic events. Have you ever experienced this kind of event as a physician?" Participants who responded "no" to this question were deemed to have a negative screen (a score of 0) and were not prompted to answer the next five questions that assess for probable PTSD. Participants who responded "yes" to the workplace trauma exposure question completed the second part of the scale, which consists of five questions assessing PTSD symptoms in the last month ("no" = 0; "yes" = 1) for total score of 0 to 5 points, with a cutoff score of 3 determining a likely PTSD diagnosis with a sensitivity of 95% and specificity of 85%.[5]

© 2023 Ptak MK et al. *JAMA Network Open.*

eAppendix 2. Survey Weights Strategy

Post-stratification and attrition weights were calculated in accordance with previously described methods.[7]

Post-stratification weights

We obtained data of US first-year resident physicians starting their training in 2018 and 2019 from the American Association of Medical Colleges (AAMC) to use as our target population. The data included demographic information (i.e., gender, race and ethnicity) of the overall population of first-year resident physicians and by residency specialty. Specialties were coded as surgical or non-surgical using the American College of Surgeons classification.[8] Within each specialty group (i.e., surgical vs. non-surgical), we obtained the number of female and male and the number of Asian, White, and underrepresented minority first-year residents. We then used the R package "anesrake"[9] to generate post-stratification weights in a 3-step process. First, we generated between-cohort weights (w1b) with cohort year as the raking variable. Second, we generated weights (w1wa) with specialty within each cohort as the raking variables. Third, we generated weights (w1wb) with the raking variables being gender and race within each specialty group (surgical and non-surgical) in each cohort.

Attrition weights

Among the 3,814 residents who completed the baseline survey, 1,957 (51.3%) completed the PC-PTSD-5 at the end-of-year survey. Using LASSO regression, we assessed which baseline characteristics significantly predicted completion of the PC-PTSD questionnaire at month 12 of residency among participants who enrolled in the baseline survey. Three variables were identified and therefore included in the estimation of attrition weights: gender (male vs. female), race/ethnicity (underrepresented in medicine vs. non-underrepresented in medicine), and specialty group (surgical vs. non-surgical). We then used the R package "twang"[10] to employ gradient-boosted models to estimate the propensity score of PC-PTSD-5 completion (p) and extracted attrition weights (w2) from the propensity score using the R function "get.weights". The attrition weight for each participant that we included in the analysis is 1 divided by p, where p is the propensity score.

© 2023 Ptak MK et al. *JAMA Network Open.*

Total scaled weights

After obtaining post-stratification and attrition weights, we calculated the total weights as follows: Total_weights = w1b * w1wa * w1wb * w2. Then, we scaled the total weights so that the weighted totals would add to the true sample size by using the formula "Total_Scaled_Weights = Sample_Size * Total weights/sum(Total weights)". The “svdesign” function of the R package “survey”[11,12] was used to incorporate the generated survey weights into the present study dataset.

© 2023 Ptak MK et al. *JAMA Network Open.*

eReferences

1. Sen S, Kranzler H, Krystal JH, Speller H, Chan G, Gelernter J, Guille C. A prospective cohort study investigating factors associated with depression during medical internship. *Arch Gen Psychiatry*. 2010;67(6):557-65.

2. Felitti VJ, Anda RF, Nordenberg D, et al. Relationship of childhood abuse and household dysfunction to many of the leading causes of death in adults. The Adverse Childhood Experiences (ACE) Study. *Am J Prev Med*. 1998;14(4):245-258.

3. Costa PT Jr, McCrae RR. Stability and change in personality assessment: the revised NEO Personality Inventory in the year 2000. *J Pers Assess*. 1997;68(1):86-94.

4. Seelig CB, DuPre CT, Adelman HM. Development and validation of a scaled questionnaire for evaluation of residency programs. *South Med J*. 1995;88(7):745-750.

5. Prins A, Bovin MJ, Smolenski DJ, et al.. The primary care PTSD screen for DSM-5 (PC-PTSD-5): development and evaluation within a veteran primary care sample. *J Gen Intern Med*. 2016;31(10):1206-1211.

6. Vance MC, Mash HBH, Ursano RJ, et al. Exposure to Workplace Trauma and Posttraumatic Stress Disorder Among Intern Physicians. *JAMA Netw Open*. 2021;4(6):e2112837.

7. Fang Y, Bohnert ASB, Pereira-Lima K, et al. Trends in Depressive Symptoms and Associated Factors During Residency, 2007 to 2019: A Repeated Annual Cohort Study. *Ann Intern Med.* 2022;175(1):56-64.

8. American College of Surgeons. What are the surgical specialties? ACS. Accessed January 16, 2023. https://www.facs.org/for-medical-professionals/education/online-guide-to-choosing-a-surgical-residency/guide-to-choosing-a-surgical-residency-for-medical-students/faqs/specialties/

9. DeBell M. Best Practices for Creating Survey Weights. In: Vannette DL, Krosnick JA, eds. *The Palgrave Handbook of Survey Research*. Springer International Publishing; 2018:159-162.

10. Griffin BA, Ridgeway G, Morral AR, Burgette LF. Toolkit for weighting and analysis of nonequivalent groups (TWANG) website. RAND Corporation; 2014. Accessed January 16, 2023. https://www.rand.org/statistics/twang

11. Lumley T. Analysis of complex survey samples. *J Stat Softw*. 2004;9(8):1-19.

12. Lumley T. *Complex Surveys: A Guide to Analysis Using R*. eBook version. John Wiley & Sons; 2011.

13. Association of American Medical Colleges. Underrepresented in medicine definition. Accessed January 15, 2023. https://www.aamc.org/what-we-do/equity-diversity-inclusion/underrepresented-in-medicine

© 2023 Ptak MK et al. *JAMA Network Open.*
