# Supplementary material for: Posttraumatic Stress Disorder Symptoms Among First-Year Resident Physicians Working Before and During the COVID-19 Pandemic
Source: JAMA Netw Open. 2023 Aug 22;6(8):e2330241. doi: 10.1001/jamanetworkopen.2023.30241 (PMC10445197; doi:10.1001/jamanetworkopen.2023.30241)
Supplement: Supplement 2. — Data Sharing Statement [file jamanetwopen-e2330241-s002.pdf]

Data Sharing Statement

Ptak. Posttraumatic Stress Disorder Symptoms Among First-Year Resident Physicians Working Before and During the COVID-19 Pandemic. *JAMA Netw Open*. Published August 22, 2023. doi:10.1001/jamanetworkopen.2023.30241

Data
Data available: Yes
Data types: Deidentified participant data
How to access data: Data are identified through ICPSR:
https://www.openicpsr.org/openicpsr/project/129225/version/V1/view
When available: With publication

Supporting Documents
Document types: Informed consent form
How to access documents: pereiral@umich.edu
When available: With publication

Additional Information
Who can access the data: researchers whose proposed use of the data has been approved
Types of analyses: specified purpose
Mechanisms of data availability: Deidentified data will be publicly available via ICPSR
https://www.openicpsr.org/openicpsr/project/129225/version/V1/view
